# Early consolidation of development and physiology of an identified presynaptic nerve terminal

**DOI:** 10.1186/1471-2202-14-124

**Published:** 2013-10-17

**Authors:** Matthew Laviolette, Bryan A Stewart

**Affiliations:** 1 Department of Biology, University of Toronto Mississauga, 3359 Mississauga Rd, Mississauga, ON L5L 1C6, Canada

## Abstract

**Background:**

A central objective in the field of neurobiology is to understand the developmental plasticity of neurons. The pursuit of this objective has revealed the presence of critical periods in neural development. Here, critical periods are defined as developmental time windows during which neural remodeling can take place; outside of these times neural plasticity is reduced. We have taken advantage of transgenic technology at the *Drosophila melanogaster* neuromuscular junction (NMJ) to investigate developmental plasticity and critical period determination of an identifiable nerve terminal.

**Results:**

Using temperature-dependent Gal80 control of transgene expression, we regulated the expression of dNSF2^E/Q^, a dominant-negative version of the *Drosophila* NSF2 gene, by shifting developing embryos and larvae between permissive and restrictive temperatures. dNSF2^E/Q^ reduces synaptic strength and causes tremendous overgrowth of the neuromuscular junctions. We therefore measured synaptic transmission and synaptic morphology in two temperature-shift paradigms. Our data show that both physiological and morphological development is susceptible to dNSF2^E/Q^ perturbation within the first two days.

**Conclusion:**

Our data support the view that individual motor neurons in *Drosophila* larvae possess a critical window for synapse development in the first one to two days of life and that the time period for morphological and physiological plasticity are not identical. These studies open the door to further molecular genetic analysis of critical periods of synaptic development.

## Background

As the field of neuroscience has advanced it has become increasingly well understood that many neural circuits of the brain have non-linear developmental profiles; that is, there are distinct time windows during which the effects of activity on development are particularly strong and long lasting [1]. These times are known as critical periods in development. Experience during critical periods modifies neural circuit architecture and behavior in fundamental ways that become highly stable and therefore permanent [2]. Once a critical period in development has ended subsequent experience generally has little impact on the organization of the neural circuit involved [3,4]. The importance of critical periods reach particular significance when there has been a disruption to the normal course of development during one of these periods. Thus, identification of critical periods and the mechanisms that underlie them is of great interest.

Several model systems have been utilized to investigate critical periods in development. The classic model for critical period research has long been development of ocular dominance columns in the striate cortex of cats and other mammals [5-10]. Other models studied in detail include auditory space mapping in barn owls [11], connectivity in the auditory pathway [12], filial imprinting in ducks [13], and song learning in birds [14-17]. While progress is being made to understand critical period phenomena as they pertain to complex vertebrate systems, the present study was undertaken to investigate developmental plasticity and critical period determination at single synaptic connections of identifiable motor neurons in the model genetic system *Drosophila melanogaster.*

The chief aim of this study was to test the hypothesis that during development of the *Drosophila* larval neuromuscular junction there are important developmental periods during which morphological and physiological phenotypes of the synapse are consolidated. To test this hypothesis we employed conditional expression of a transgene known to disrupt both synaptic development and physiology with two experimental approaches. First, we asked: when does the NMJ become stable if it is faced with a disruptive cue following an initial period of normal growth? Second, we asked: what is the capacity of the neuromuscular junction to return to normal following an initial disruption in development?

We previously described presynaptic overgrowth caused by the neural expression of a dominant-negative version of N-ethylmaleimide sensitive factor (NSF^E/Q^) [18] and confirmed that this phenotype appears early in development and is consistent with genetic loss-of-function NSF alleles [19].

While the precise mechanism by which this transgene causes overgrowth is still under investigation [20], here we have used it as a tool to perturb synaptic development at different periods of larval life. The transgene is expressed with the well-established UAS-Gal4 system [21]; Gal4 is a yeast transcriptional activator that will cause transcription of genes that carry the Gal4 Upstream Activation Sequence (UAS). We previously engineered the *Drosophila* NSF2 gene to carry the dominant negative point glutamate –glutamine mutation within the NSF2 ATPase domain for use in the GAL4-UAS system (UAS-dNSF2^E/Q^) [18]. When expressed specifically in neurons this transgene causes disruption of both synaptic morphology and physiology, observed primarily as a massive overgrowth of the NMJ accompanied by severe reduction in the strength of synaptic transmission.

To render UAS-dNSF2^E/Q^ expression conditional, we made use of Gal80, a yeast repressor of Gal4. A temperature-sensitive mutation of Gal80 (Gal80^ts^) leaves the Gal80 protein functional at room temperature but inactive at high temperature. Thus, at room temperature Gal80 represses Gal4, but at high temperature Gal80^ts^ no longer represses Gal4 and Gal4-dependent transcription proceeds. *Drosophila* carrying a ubiquitously expressed Gal80^ts^ have been previously described [22] and we have used them here in combination with UAS-Gal4 dependent expression of dNSF2^E/Q^ to regulate UAS-dNSF2^E/Q^ gene transcription through development.

## Results

### Synaptic morphology

In order to carry out the proposed experiments we first sought to validate our approach. We have previously shown that expression of *UAS-dNSF2*^*E/Q*^ causes NMJ overgrowth [18]; we therefore sought to establish that Gal80^ts^ enables temperature-dependent control of the dNSF2^E/Q^ phenotype. We predicted that when *Gal80*^*ts*^*; elav- Gal4::UAS-dNSF2*^*E/Q*^ larvae are raised at room temperature Gal80^ts^ should repress Gal4 and the NMJs should appear normal, while if larvae are raised at 30°C, Gal80^ts^ is not active and Gal4 is active, and the dNSF2^E/Q^ phenotype should appear. We evaluated these two possibilities.

**Figure 1:**
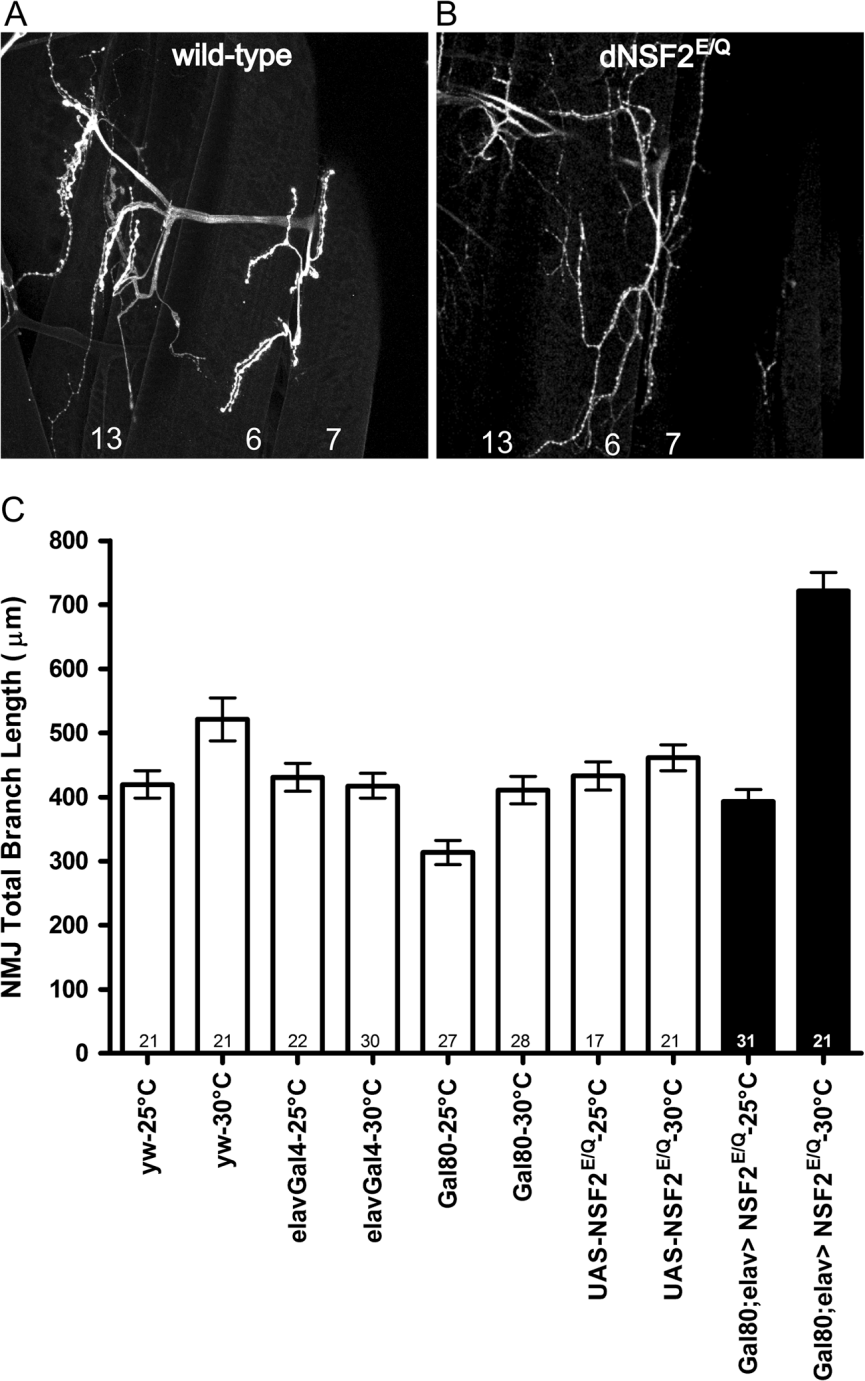
**Gal80 control of Gal4 dependent UAS-dNSF2**^**E/Q **^**morphological phenotypes.** Typical immunofluorescent images of **(A)** wild-type and **(B)** elavGal4 > UAS-dNSF2^E/Q^ Drosophila larval neuromuscular junctions (NMJ). Muscle 6, 7, and 13 are labeled. The dNSF2^E/Q^ neuromuscular junctions are significantly overgrown compared to wild-type. **(C)** Total NMJ length was measured and quantified for the indicated genotypes from larvae raised continuously at either 25° or 30°C. At 25°C the presence of Gal80 suppresses the elavGal4 > UAS-dNSF2^E/Q^ driven overgrowth, whereas at 30°C the NMJs remain overgrown (black bars).

First, we compared NMJ morphology of *GAL80*^*ts*^*; elav- Gal4::UAS-dNSF2*^*E/Q*^ to that of *yw*, Gal80, Gal4 and UAS control larvae when they were raised at room temperature. The NMJs in all of these genotypes appeared wild-type, with no significant difference observed in the total NMJ length among them (Figure 1, p > 0.05, ANOVA). This indicates that Gal80 is effectively suppressing Gal4-dependent transcription.

Next we raised larvae of all of these genotypes at 30°C and carried out a similar analysis of NMJ morphology. The main observation we made is that NMJs in the *GAL80*^*ts*^*; elav- Gal4::UAS-dNSF2*^*E/Q*^ now appeared significantly overgrown, similar to what we have previously published, and substantially longer than any of the other control genotypes we examined (Figure 1). There was no detectable difference in NMJ length between any of the control lines when they were reared in the higher temperature. The mean NMJ length in *GAL80*^*ts*^*; elav-Gal4::UAS-dNSF2*^*E/Q*^ at 30°C was not significantly different from that found in *elav-Gal4::UAS-dNSF2*^*E/Q*^ larvae at 30°C, and such NMJs were both significantly longer than the controls. Thus, in the high temperature conditions, Gal80 activity is repressed and Gal4-dependent transcription proceeds yielding the expected dNSF2^E/Q^ phenotype.

Altogether, these initial experiments indicate that GAL80^ts^ enables temperature-dependent control of the NMJ overgrowth phenotype caused by expression of *elav-Gal4::UAS-dNSF2*^*E/Q*^. We next set out to test whether changing the rearing temperature of the larvae at different developmental time points led to alterations in synaptic development.

We probed developmental plasticity in two ways: we either allowed the larvae to start developing normally (at 25°C, Gal4-dependent dNSF2^E/Q^ suppressed) and then shifted them to 30°C (Gal4-dependent dNSF2^E/Q^ active) or we did the reverse and started larvae growing at 30°C and then shifted them to 25°C and inhibiting dNSF2^E/Q^ at later time points.

**Figure 2:**
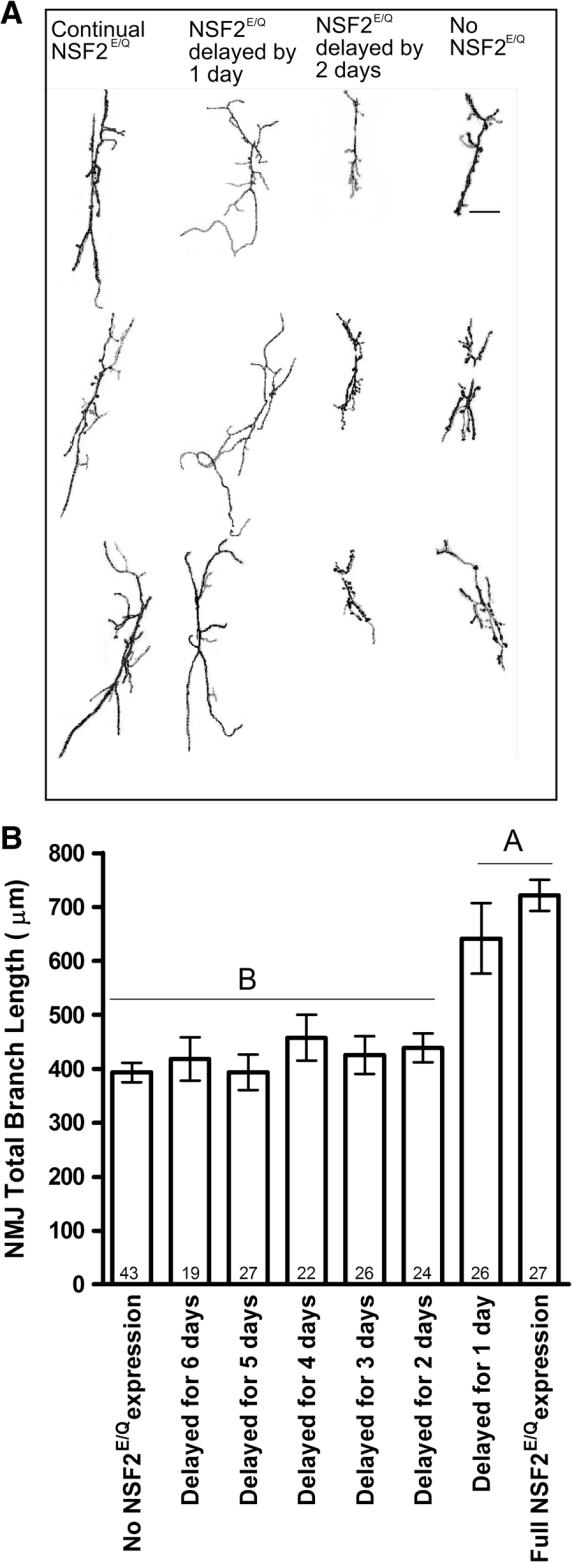
**Delayed expression of UAS-dNSF2**^**E/Q**^**.** Larvae were initially reared at 25°C (Gal4 off) and then shifted to 30°C (Gal4 on) and the NMJs were measured at the wandering third larval instar. **(A)** 3 representative NMJs from each of the rearing conditions indicated. Scale bar is 10 μm. **(B)** Summary of NMJ length measurements from each of the rearing conditions. The bars represent mean ± SEM with the number of NMJs measured within each bar.

In the first set of experiments we delayed the expression of dNSF2^E/Q^ by allowing adult *Drosophila* to lay eggs at room temperature and allowing the larvae develop for increasing lengths of time at room temperature before moving them to 30°C (Figure 2). Thus, we let the NMJ develop normally and then challenged it with dNSF2^E/Q^ expression. We observed a very clear transition in synaptic morphology phenotypes between the second and third day of development. If eggs were laid at room temperature and embryos/larvae developed for only 1 day prior to being placed at 30°C, the NMJ of those larvae at the third instar were not distinguishable from larvae that had been raised entirely at 30°C; that is they appeared mutant. In contrast, if the eggs were laid at 25°C and allowed to develop for 2 days prior to being shifted to 30°C, the NMJs of those larvae at third instar were indistinguishable from larvae that had been raised entirely at 25°C; that is they appeared wild-type. Thus, there appears to be a consolidation of synaptic development between 24 and 48 hrs after egg laying.

**Figure 3:**
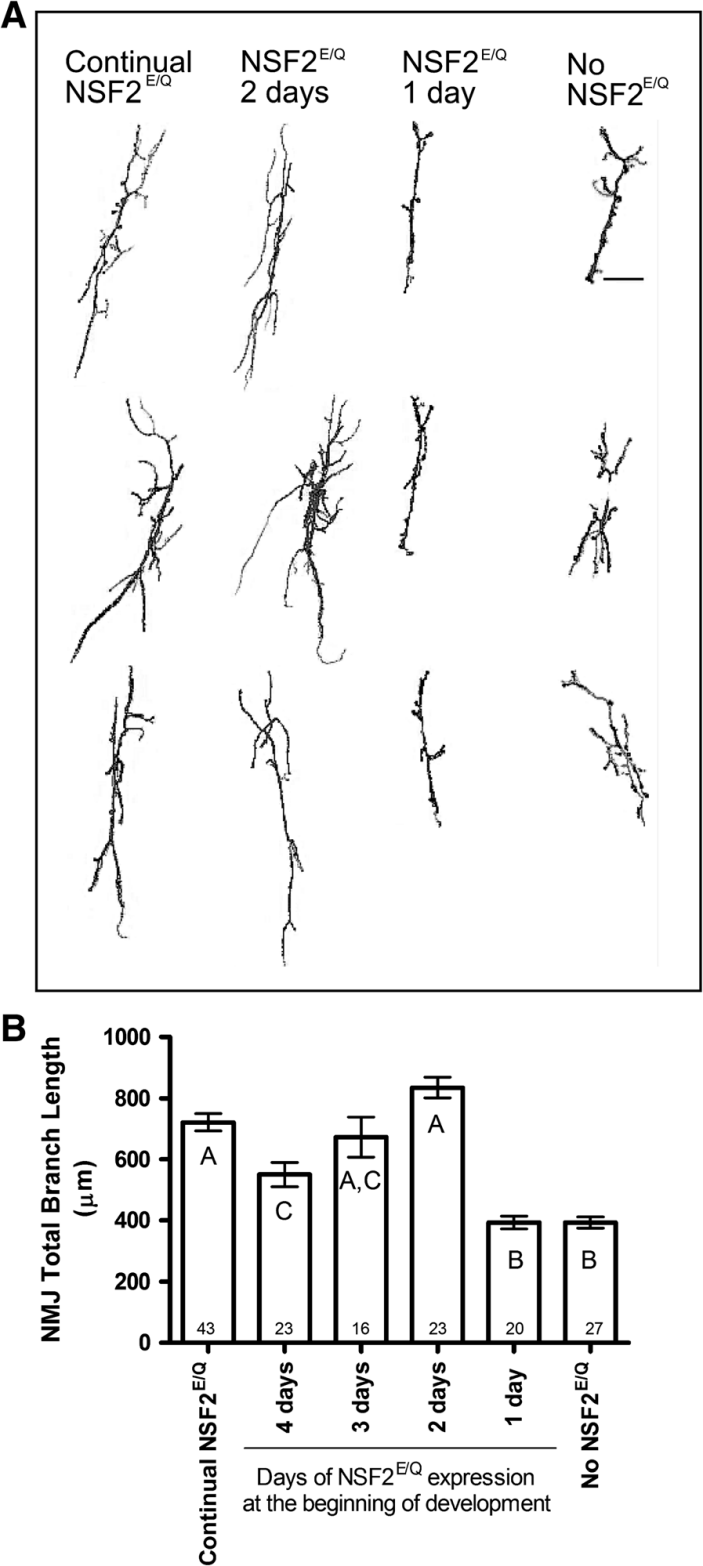
**Shortened expression of UAS-dNSF2**^**E/Q**^**.** Larvae were initially reared at 30°C (Gal4 on) and then shifted to 25°C (Gal4 off) and the NMJs were measured at the wandering third larval instar. **(A)** 3 representative NMJs from each of the rearing conditions indicated. Scale bar is 10 μm. **(B)** Summary of NMJ length measurements from each of the rearing conditions. The bars represent mean ± SEM with the number of NMJs measured within each bar.

We next carried out the experiment with reversed temperature shifts: adults laid eggs at 30°C and the embryos and larvae developed at this temperature, with Gal4 active, for fixed intervals before being moved to 25°C (Figure 3). In doing so we expected the NMJs to start developing a mutant phenotype.

We observed that if larvae expressed dNSF2^E/Q^ for at least the first two days of development that they showed dNSF2^E/Q^ mutant phenotype. If they were exposed to dNSF2^E/Q^ for only the first day and then shifted to 25°C, they developed a normal synaptic phenotype at the third instar. The data gathered with this temperature regime were more variable than that of the first experiment but they data further support the notion that there is a critical developmental time period in the first 24–48 hours. If the mutant-inducing transgene expression is removed by 24 hours, the synapse develops normally. If transgene expression continues for the first 48 hours or beyond, the synapse will develop with a mutant phenotype, even if the transgene is later suppressed.

### Synaptic physiology

In parallel with the above experiments on synaptic morphology we carried out electrophysiological studies of synaptic transmission to determine if there were critical periods of physiological development of the synapse. We have previously shown that expression of dNSF2^E/Q^ results in significant impairment of synaptic transmission which is manifest by reduced amplitude of nerve evoked excitatory junctional responses, as well as reduced frequency of spontaneously released miniature excitatory junctional potentials. Using the same temperature-shift paradigm as above, we evaluated the effect of dNSF2^E/Q^ on synaptic transmission.

**Figure 4:**
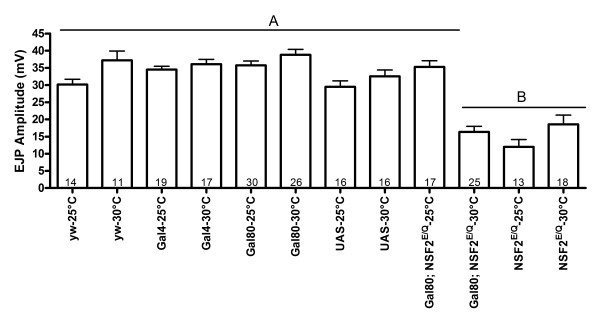
**Gal80 control of Gal4 dependent UAS-dNSF2**^**E/Q **^**physiological phenotypes.** Excitatory Junctional Potentials (EJPs) were recorded from Muscle 6 and their amplitudes were measured. The dNSF2^E/Q^ neuromuscular junctions are significantly overgrown compared to wild-type. When Gal80; elavGal4 > UAS-dNSF2^E/Q^ larvae were raised at 25°C their EJP amplitude was comparable to that of all the control genoptypes. When these larvae were raised 30°C their EJPs were significantly smaller, comparable to the mutant, elavGal4 > UAS-dNSF2^E/Q^ phenotype (black bars). The bars represent mean ± SEM EJP amplitude (mV) with the number of muscles recorded indicated within each bar.

Firstly, we verified that we could observe temperature dependent alteration of synaptic transmission in the *GAL80*^*ts*^*; elav-Gal4::UAS-dNSF2*^*E/Q*^ larvae. When larvae of this genotype were raised at 25°C their EJPs were about 40 mV in amplitude and indistinguishable from the EJPs of *yw*, Gal80, Gal4 and UAS control larvae raised at 25°C. In contrast, when *GAL80*^*ts*^*; elav-Gal4::UAS-dNSF2*^*E/Q*^ larvae were raised continuously at 30°C their EJPs were significantly smaller, averaging about 15 mV and these were not distinguishable from those in *elav-Gal4::UAS-dNSF2*^*E/Q*^ larvae raised at either 30°C or 25°C (Figure 4). Furthermore, there was no difference in muscle fiber membrane potential or input resistance (Table 1) among the treatments which could account for these changes in EJP amplitude.

**Table 1:** Electrophysiological parameters measured from control groups at 25°C and 30°C

	***yw***		***Elav-Gal4***		***Gal80***^***ts***^	
	**25°**	**30°**	**25°**	**30°**	**25°**	**30°**
	^**n=14**^	^**n=11**^	^**n=17**^	^**n=17**^	^**n=30**^	^**n=26**^
Vm (mV)	-66.3 ± 1.5	-69.7 ± 2.0	-63.8 ± 0.7	-65.6 ± 1.1	-68.3 ± 0.9	-66.1 ± 0.8
Rm (MΩ)	7.6 ± 0.4	11.6 ± 1.4	10.5 ± 0.8	9.6 ± 0.6	8.1 ± 0.7	9.7 ± 0.6
mEJP (mV)	0.8 ± 0.02	1.0 ± 0.08	1.0 ± 0.04	0.8 ± 0.05	1.0 ± 0.07	0.8 ± 0.05
mEJP (Hz)	2.4 ± 0.2	0.6 ± 0.1	3.1 ± 0.3	0.4 ± 0.06	1.1 ± 0.06	1.3 ± 0.3
	***UAS-dNSF2***^***E/Q***^		***Gal80***^***ts***^ ***;UAS-dNSF2***^***E/Q***^		***Elav-Gal4;Gal80***^***ts***^ ***; UAS-dNSF2***^***E/Q***^	
	**25°**	**30°**	**25°**	**30°**	**25°**	**30°**
	^**n=16**^	^**n=15**^	^**n=17**^	^**n=25**^	^**n=13**^	^**n=18**^
Vm (mV)	-67.44 ± 1.1	-68.9 ± 1.2	-69.9 ± 1.4	-63.8 ± 0.6	-65.1 ± 1.2	-67.4 ± 1.3
Rm (MΩ)	11.7 ± 0.8	11.2 ± 0.8	8.2 ± 0.8	12.9 ± 0.8	7.2 ± 0.8	7.7 ± 0.7
mEJP (mV)	1.2 ± 0.05	1.3 ± 0.08	1.2 ± 0.09	0.7 ± 0.04	0.7 ± 0.05	0.7 ± 0.04
mEJP (Hz)	2.0 ± 0.1	1.3 ± 0.2	2.0 ± 0.2	0.8 ± 0.1	1.1 ± 0.3	0.7 ± 0.1

Vm, resting membrane potential; Rm, muscle fiber membrane resistance; mEJP, miniature excitatory junction potential.

We also made observations on the spontaneous release of neurotransmitter (Table 1). The amplitudes of the mEJPs were relatively consistent across the genotypes under the two temperature conditions; dNSF2^E/Q^ expression did not alter mEJP amplitude and likewise *GAL80*^*ts*^*;elav-Gal4::UAS-dNSF2*^*E/Q*^ larvae raised continuously at 25°C were not different from any of the other control lines at 25°C. We did note however a small but significant reduction in mEJP amplitude when *GAL80*^*ts*^*;elav-Gal4::UAS-dNSF2*^*E/Q*^ larvae were raised at 30°C compared to 25°C.

The mEJP frequency data were more variable among genotypes and at different temperatures. We previously reported a decline in mEJP frequency in *elav-Gal4::UAS-dNSF2*^*E/Q*^ larvae and we also report here that *GAL80*^*ts*^*;elav-Gal4::UAS-dNSF2*^*E/Q*^ larvae raised at 30°C had a reduced mEJP frequency compared to those raised at 25°C. However, we also noted a temperature-dependent decline in mEJP frequency in the *yw,* elav-Gal4, and UAS-dNSF2 strains. Thus, in this data set it is difficult to ascribe the decline in mEJP frequency in the *GAL80*^*ts*^*;elav-Gal4::UAS-dNSF2*^*E/Q*^ larvae solely to activity of *UAS-dNSF2*^*E/Q*^.

**Figure 5:**
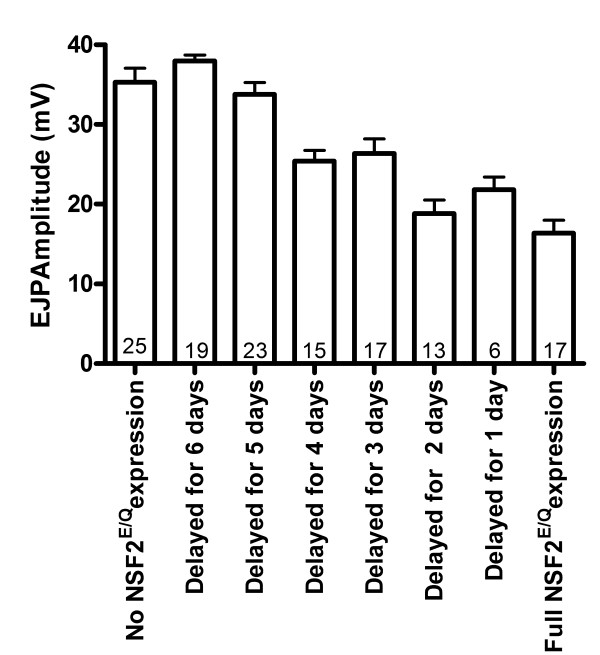
**Effect of delaying UAS-dNSF2**^**E/Q **^**on synaptic physiology.** EJP amplitudes were measured from larvae initially reared at 25°C (Gal4 off) and then shifted to 30°C (Gal4 on). If expression is only delayed for 1 or 2 days, EJP amplitudes are small and comparable to full continuous UAS-dNSF2^E/Q^ expression. If expression is delayed by 3 days or more, the EJP amplitudes return to normal levels. The bars represent mean ± SEM EJP amplitude (mV) with the number of muscles recorded indicated within each bar. Letters within the bars represent statistically similar groups determined with ANOVA (p < 0.05) and the Dunnett *post hoc* comparison of all conditions to full UAS-dNSF2^E/Q^ expression.

Having established Gal80-dependent control of the dNSF2^E/Q^ physiological phenotype, we next measured synaptic transmission from larvae that started life at room temperature and were later shifted to high temperature during embryonic/larval development; thus dNSF2^E/Q^ expression was delayed (Figure 5). In this experiment, we found that there was a general trend to smaller EJP amplitudes the more time the larvae spent at 30°C in their early life. We analyzed these data with a linear regression of the data points and found the best fit line had a slope of −3.1 ± 0.45 mV/day (r^2^ = 0.89). ANOVA testing further showed a significant variance among the data (p < 0.0001) and Dunnett’s multiple comparison post-hoc test was used to compare no dNSF2^E/Q^ expression to the other groups. There was no statistical difference in the mean EJP amplitude for larvae in which dNSF2^E/Q^ expression was delayed for 6 or 5 days compared to no dNSF2^E/Q^ expression. However, if dNSF2^E/Q^ expression was delayed by for 4 days or less, then the EJP amplitudes were statistically smaller in comparison to no dNSF2^E/Q^ expression.

**Figure 6:**
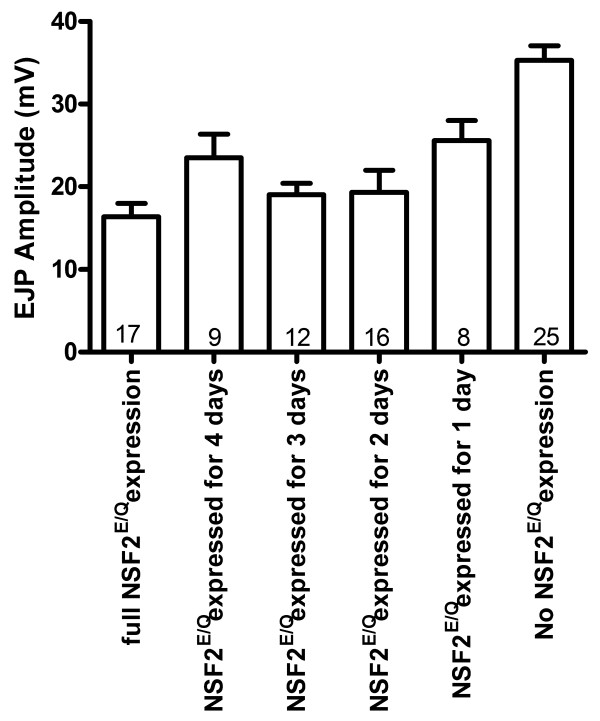
**Effect of shortened UAS-dNSF2**^**E/Q **^**expression on synaptic physiology.** Larvae were initially reared at 30°C (Gal4 on) and then shifted to 25°C (Gal4 off) and the EJP amplitudes were measured at the third instar stage. If larvae were exposed to 25°C for the 2–4 days prior to emerging as third instar larvae (expressed for 1 day group), their EJPs were indistinguishable from larvae who were raised entirely at 30°C (no expression group). Letters within the bars represent statistically similar groups determined with ANOVA (p < 0.05) and the Dunnett *post hoc* comparison of all conditions to full UAS-dNSF2^E/Q^ expression. When reared at 30°C larvae reached the third instar stage sooner and so there are fewer days in the analysis.

We again carried out the reversed temperature shift experiments with the adult flies laying eggs at 30°C (dNSF2^E/Q^ active) and allowing the embryos and larvae developed at this temperature for fixed intervals before being moved to 25°C and allowing Gal80 to suppress Gal4. Recordings of synaptic strength from larval NMJs of such larvae revealed that if dNSF2^E/Q^ was active the first 2, 3, or 4 days AEL then the EJP amplitude was not distinguishable from full dNSF2^E/Q^ activity (Figure 6). However, if dNSF2^E/Q^ was only active for the first day AEL then EJPs were larger than those observed under full dNSF2^E/Q^ activity but still smaller than EJPs recorded with no dNSF2^E/Q^ activity. In other words, dNSF2^E/Q^ activity induced a long-lasting reduction in synaptic strength if it was expressed for the first 2 days AEL; if it was expressed for only the first day AEL a partial suppression was observed. These data indicate there is a time-sensitive window of physiological development in the first two days AEL.

### Co-dependence of physiology and morphology

We further analyzed our data by combining our morphometric and physiology data sets to examine co-dependent features of dNSF2^E/Q^ activity and the sensitivity of the developmental changes we observed. We did not gather pairwise data for each morphometric and physiologic parameter we measured (i.e. we did not measure NMJ length and EJP amplitude for the same NMJ) so we therefore plotted our sampled data as NMJ length vs. EJP amplitude for each of the rearing conditions studied.

**Figure 7:**
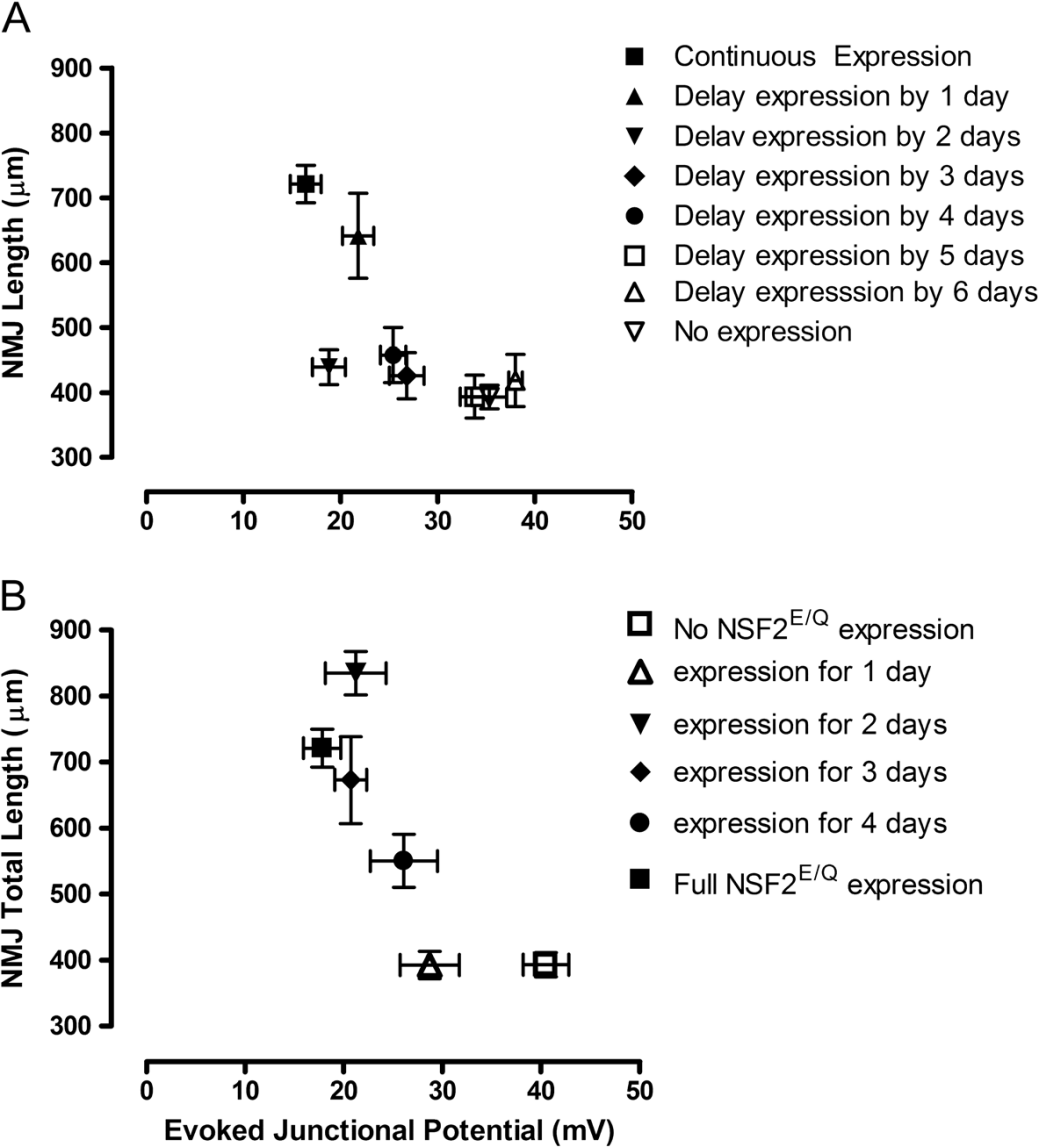
**Correlation of morphological and physiological associated with temporal expression of UAS-dNSF2**^**E/Q **^**during (A) delayed expression and (B) shortened expression protocols.** This analysis shows there is a non-linear relationship between synaptic physiology and morphology.

We first plotted these data for the rearing temperature schedule in which dNSF2^E/Q^ activity was delayed (Figure 7A). On this graph, we observe that if dNSF2^E/Q^ activity is delayed to the 5 or 6^th^ day AEL there is essentially no difference between the dNSF2^E/Q^ expressing samples and the non-expressing dNSF2^E/Q^ samples for both NMJ length and EJP amplitude. However, as dNSF2^E/Q^ activity starts to occur earlier in development we see that while NMJ length remains fairly constant at about 400 μm, the EJP amplitude starts to get smaller, going from ~40 mV down to a range near ~20-25 mV. Thus, it appears that during the 2^nd^, 3^rd^, and 4^th^ day AEL synaptic physiology is sensitive to dNSF2^E/Q^ activity whereas NMJ morphology is not. Finally, when dNSF2^E/Q^ activity is delayed by only one day both EJP amplitudes and NMJ morphology appear quite similar to those observed under full dNSF2^E/Q^ activity; that is the NMJs are substantially overgrown and EJPs significantly smaller.

Using the same graphical analysis for the reverse temperature regimen also reveals an interesting pattern. In this series, dNSF2^E/Q^ is initially active but then later reduced. We see that if dNSF2^E/Q^ is active continuously, or active for the first 2 or 3 days AEL, the data fall into roughly the same group with overgrown NMJs and small EJPs. If dNSF2^E/Q^ is only active for the very first day AEL, NMJ length is comparable to no dNSF2^E/Q^ activity, whereas EJP amplitude seems somewhat more sensitive, showing smaller EJPs at this data point (Figure 7B).

Altogether this graphical analysis indicates that the NMJ morphology is fairly robust and resistant to disturbance by dNSF2^E/Q^; for the most part morphology appears as normal or mutant with little intermediate phenotype. In contrast, synaptic physiology seems more sensitive to dNSF2^E/Q^ activity and it does show phenotypes intermediate to full dNSF2^E/Q^ activity and no dNSF2^E/Q^ activity.

## Discussion

The chief aim of this study was to test the hypothesis that there is a developmental period of the *Drosophila* larval neuromuscular junction during which synaptic morphological and physiological phenotypes are consolidated. We tested this idea using conditional expression of a transgene that was previously shown to disturb both the development and the physiology of this synaptic connection. We observed that there are indeed sensitive periods of synaptic development during the first one to two days of larval life, but that synaptic physiology seems to be more sensitive to disturbance than synaptic morphology. These are important data that extend our knowledge of critical periods of neural development to the level of synapses of identifiable neurons in a genetically tractable model system.

The idea of a critical period in neural development is a central tenet in the field of neurobiology. The classic work of Lorenz in the behavioral realm and Hubel and Wiesel on the development of ocular dominance columns are the first instances in the larger field of neural plasticity, which includes synaptic competition and synaptic refinement [23]. Indeed, plasticity in the visual system has been one of the most extensively studied models of critical periods in development, although critical periods have been observed in a wide variety of mammalian sensory systems [1]. Analysis of the mouse NMJ has shown activity-dependent synaptic competition to establish the mature NMJ [24,25] but there are relatively fewer studies of critical periods on the motor side of the nervous system.

Rarer are studies on critical periods in neural development of invertebrates. Axonal pruning during development within the CNS of *Drosophila*, particularly the olfactory system and the mushroom bodies, provide some examples [26-29] although this development has not strictly been shown to have activity-dependent critical periods. Additionally, studies on neural rearrangements during insect metamorphosis are informative in this regard, having a strongly-timed developmental component [30,31]. An interesting description of critical periods within the mechanosensory system of *C. elegans* using a sensory deprivation design [32] illustrates the likelihood of critical periods in invertebrate sensory systems.

A useful comparator study is that of Jarecki and Keshishian [33]. They examined the role of neural activity in synaptic development, using synaptic target selection as their measurement. The *Drosophila* NMJ is characterized by precise muscle target selection by the innervating motor neurons. Jarecki and Keshishian observed that the occurrence of ectopic targeting can be influenced by manipulating neural activity; reducing activity leads to an increase in the appearance of inappropriate synaptic connections. Importantly, they showed that manipulations performed during embryogenesis and the first larval instar was important to observe the ectopic synapses, thus indicating a sensitive period in synaptic target selection during this early time window. Their observation is largely in congruence with our results, albeit using a different molecular perturbation; we have additionally added the profile of native NMJ target neurons and the physiological changes to this early critical period in *Drosophila* NMJ development.

Although our results are quite clear that there is a sensitive time window during early development, our interpretation of these data are somewhat limited. Firstly, the primary cause of the synaptic phenotypes induced by dNSF2^E/Q^ is not well understood. While NSF is well known as an ATPase that disassembles the SNARE complex following vesicle fusion, NSF has been increasingly shown to have non-SNARE roles in the cell [34,35]. Indeed, we have shown that expression of dNSF2^E/Q^ is associated with a variety of phenotypes at this NMJ for which one would not normally invoke SNARE disassembly as a cause. Such phenotypes include alteration of the synaptic actin cytoskeleton and impaired synaptic vesicle dynamics [36]. Our earlier genetic studies [37,38] also identified a variety of genes that interact with dNSF2^E/Q^ NMJ overgrowth, including genes that encode cytoskeletal elements, transcription factors, and signaling proteins. While the present study does not shed light on the phenotypic action of dNSF2^E/Q^, the transgene has proven to be a useful tool for dissecting sensitive developmental time windows.

Additionally, although we have demonstrated control of the synaptic phenotypes using the Gal4 / Gal80 competition approach, the use of temperature to regulate a temperature sensitive mutation of Gal80 limits our ability to more precisely define when the critical period starts and ends. Use of other tools such as the Geneswitch system [39] or the newer Q system [40] may help in this regard.

With these limitations in mind, it is useful to project the types of synaptic changes that may occur during the consolidation process. It is particularly interesting that it appears that once the NMJ starts to develop under normal conditions, it continues to develop normally even in the face of a perturbing factor. This implies that the cytoskeletal and cell adhesion mechanisms necessary for proper synaptic architecture are present at the required levels and resistant to the changes induced by dNSF2^E/Q^. Alternatively, repair mechanisms that counteract the action of dNSF2^E/Q^ are fully established in the first two days of synaptic development. Delineating between these possibilities will be the subject of future studies.

## Conclusion

In summary, we have demonstrated that the morphological and physiological development of the *Drosophila* larval neuromuscular junction is sensitive to perturbation by transgenic gene expression during the first one to two days after egg laying. Activation of the perturbing transgene in subsequent days reveals greater and greater resistance to phenotypic change and that the morphological characteristics of the NMJ are consolidated within 2 days, while the physiological phenotype requires an additional one or two days to become firmly established. These data represent one of the few studies on critical periods of neural development in invertebrates and of the motor systems in any species. Establishing the presence of critical period in synapse development in this genetically tractable model system will open the door to further cellular and molecular characterization of this widespread developmental phenomenon.

## Methods

### Fly stocks

*Drosophila melanogaster* stocks were raised on standard media at 25°C and/or 30°C as described in the experimental procedures. Test larvae were obtained by crossing a homozygous Gal80^ts^ (2nd chromosome) stock (McGuire et al., 2004) to a stock with a recombinant third chromosome containing: elav3A-Gal4: UAS-dNSF2^E/Q^ that has been previously described [18]. The enhancer of *elav* directs pan-neural gene expression throughout development [41]. The use of this enhancer limits the expression of the dNSF2^E/Q^ transgene to the nervous system. elav3A-Gal4: UAS-dNSF2^E/Q^ is homozygous lethal [18] and is therefore maintained over the balancer chromosome TM6, Tb. Crosses were made by collecting 20–25 virgin females placing them in a vial with 15–20 males of the appropriate genotype.

### Temperature treatments

At time zero of each experiment adults were transferred to a new vial at experimental temperature and allowed to lay eggs for 6–8 hours at 25°C or at 30°C after which the adults were removed. Such vials continued to be incubated at the starting temperature until the pre-determined day that they were transferred to the other temperature. Larvae then developed until the third instar, at which time they were removed from the vial, dissected, and processed for imaging or electrophysiology experiments.

### Dissections

The balancer chromosome TM6, Tb has an easy to score ‘tubby’ phenotype to select against ensuring that only larvae carrying the dNSF2^E/Q^ allele were chosen for dissection. Larvae were collected as third instars, placed prone on a dissecting tray and pinned down at the anterior and posterior aspects. The animals were then bathed in zero-calcium HL3 solution [42]. An incision was made along the entire dorsal aspect of the animal after which the digestive tract, ganglion system and main trachea were removed using forceps and scissors. The remaining body wall was pulled taut by pinning down all four corners of the body wall to allow for easy access and visualization of body wall muscles 6/7 in abdominal segments 3 and 4.

### Immunofluorescence and image acquisition

Following dissection, HL3 solution [42] was removed and larvae were fixed with 4% paraformaldehyde diluted in a phosphate buffered saline (PBS) solution for 10 minutes. The animals were then rinsed three times with PBS plus 0.1% Triton X-100 (PBT) solution for a total duration of thirty minutes. Next, the larvae were incubated in 1.5 ml of PBS with 1.5 μl of 1:1000 dilution FITC-conjugated goat anti-HRP (ICN Biomedical) for two hours. Following incubation the larvae were rinsed three successive times in PBT for a total duration of thirty minutes. Finally, the larvae were mounted on a slide with Vectashield (Vector Laboratories) and covered with a cover slip.

Images of the body wall muscles 6/7 in abdominal segments 3 and 4 were obtained using a Zeiss LSM 510 confocal microscope through a 20X lens and using excitation wavelength of 488 μm. Z-stack images were acquired and then projected onto a single plane for analysis using ImageJ software. Total branch length was determined by physically tracing the entire NMJ image with a cursor and then adding up the total length of all visible branches. Two or three NMJs were examined from the majority of larvae sampled although some larvae had only one NMJ examined.

### Electrophysiology

Following dissection, larvae were bathed in HL3 plus 1mM Ca^2+^. A glass recording electrode with a resistance of approximately 25 MΩ when filled with 3M KCl was used to impale the muscles and take recordings of muscle membrane potentials. Signals from the recording electrode were amplified using an Axoclamp 2B amplifier (Axon Instruments Inc. Burlingame CA) and recorded using Clampex 8.2 software. A glass stimulating electrode filled with HL3 plus 1 mM Ca2+ was used to deliver electrical current to the segmental nerve projecting to the muscles of interest. Recording were obtained from two muscles per larvae. Membrane potential recordings were analyzed using Clampfit 10.0 software. EJPs were analyzed using the cursor options of Clampfit 10.0, while mEJPs were analyzed with the template search option.

### Statistical analysis

Statistical analysis of the data was conducted using Graphpad Prism 4.0 software to perform one-way ANOVA tests in order to determine if variances between experimental groups was significant. A p value of 0.05 was chosen as the level of significance.

## Abbreviations

NMJ: Neuromuscular junction; dNSF2: Drosophila N-ethylmaleimide sensitive factor 2; EJP: Excitatory junctional potential; mEJP: Miniature excitatory junctional potential; AEL: After egg laying.

## Competing interests

The authors declare that they have no competing interests.

## Authors’ contributions

The work was conceived, designed and planned by BAS and ML. ML performed all experiments and data analysis. The text was drafted in part ML as part of an MSc thesis; the manuscript was assembled and prepared by BAS. Both authors read and approved the final manuscript.
